# The Fine-Tuned Release of Antioxidant from Superparamagnetic Nanocarriers under the Combination of Stationary and Alternating Magnetic Fields

**DOI:** 10.3390/antiox10081212

**Published:** 2021-07-28

**Authors:** Lucija Mandić, Anja Sadžak, Ina Erceg, Goran Baranović, Suzana Šegota

**Affiliations:** Ruđer Bošković Institute, 10000 Zagreb, Croatia; Lucija.Mandic@irb.hr (L.M.); Anja.Sadzak@irb.hr (A.S.); Ina.Erceg@irb.hr (I.E.); Goran.Baranovic@irb.hr (G.B.)

**Keywords:** release kinetics, superparamagnetism, stationary magnetic field, alternating magnetic fields, magnetic nanoparticles, quercetin

## Abstract

Superparamagnetic magnetite nanoparticles (MNPs) with excellent biocompatibility and negligible toxicity were prepared by solvothermal method and stabilized by widely used and biocompatible polymer poly(ethylene glycol) PEG-4000 Da. The unique properties of the synthesized MNPs enable them to host the unstable and water-insoluble quercetin as well as deliver and localize quercetin directly to the desired site. The chemical and physical properties were validated by X-ray powder diffraction (XRPD), field emission scanning electron microscopy (FE–SEM), atomic force microscopy (AFM), superconducting quantum interference device (SQUID) magnetometer, FTIR spectroscopy and dynamic light scattering (DLS). The kinetics of in vitro quercetin release from MNPs followed by UV/VIS spectroscopy was controlled by employing combined stationary and alternating magnetic fields. The obtained results have shown an increased response of quercetin from superparamagnetic MNPs under a lower stationary magnetic field and s higher frequency of alternating magnetic field. The achieved findings suggested that we designed promising targeted quercetin delivery with fine-tuning drug release from magnetic MNPs.

## 1. Introduction

Quercetin (C_15_H_10_O_7_, 3,3′,4′,5,7-pentahydroxyflavone) is a major member of the flavonols, a subclass of flavonoids, natural polyphenols [1]. It is an important component of the human’s daily diet and widely distributed in vegetables and fruits such as onions, tomatoes, berries, grapes, nuts, as well as in many flowers and leaves [2,3]. In addition, quercetin exhibits a wide range of biological and pharmacological activities, including antioxidant, anti-inflammatory, antibacterial, anti-anaemic, and anticarcinogenic activities [1,3,4,5]. Extensive studies have reported that quercetin can inhibit the proliferation of several types of cancers such as lung, prostate, breast cancer, and pancreatic tumour cells [1]. In addition, the main feature of quercetin is its antioxidant potential of OH groups in the structure that can bind to reactive oxygen species (ROS) and maintain cell viability. Quercetin has been shown to decrease the activity of antioxidant and apoptotic proteins and increase the levels of antiapoptotic proteins [6]. However, its therapeutic and clinical properties are limited due to its hydrophobic nature and low stability in the physiological medium. The problem with instability, low solubility and poor bioavailability can be successfully overcome by their loading in drug delivery systems including nanoparticles (NPs) [3,5,7,8]. The rapid growth of nanotechnology is the key to a revolutionary platform for chemical, physical, biological and mechanical properties of various materials [9,10]. There is tremendous interest in nanomaterials or NPs in the biomedical field [11]. For example, a variety of NPs is envisioned to be used in medical applications such as cancer detection, magnetic resonance imaging, cardiovascular and neurological treatment diseases, targeted drug delivery, hyperthermia, bioseparation, and gene transfer [3,9,10]. In recent years, magnetic nanoparticles (MNPs) have been highlighted among the many types of NPs [12,13,14]. Essentially, researchers were attracted by their excellent unique physical, chemical and magnetic properties [15]. In 1957, Gilchrist and coworkers showed the first use of magnetic particles for inductive heating of lymph nodes in dogs [16,17].

In 1983, Widder and coworkers reported the first use of MNPs containing doxorubicin to treat Yoshida rat sarcoma with an external magnetic field. These results represented a compelling advance in chemotherapy treatment, with complete cancer remission demonstrated in 77% of animals in the magnetically localized doxorubicin-magnetite microparticles [17,18]. MNPs have received much attention as target drugs that can replace traditional chemotherapy without the side effects [19]. Several inorganic magnetic nanoparticles (MNPs) have the potential to be used for drug delivery, but MNPs are the only magnetic materials approved by the Food and Drug Administration (FDA) for human use [3,19,20,21]. Numerous physical, biological and chemical preparation methods have been accepted for the MNP’s synthesis [22]. Magnetite NPs are most commonly used in biological applications due to their unique physicochemical properties such as particle size, size distribution, shape and high surface area [23,24]. They exhibit interesting properties such as superparamagnetism, high field irreversibility and high saturation field [24,25,26]. Due to these properties, the superparamagnetic NPs can become magnetized when the external magnetic field is used and they do not remain magnetized when the field is turned off. The localization of drugs with MNPs in combination with an external magnetic field and their retention until the completion of therapy [26] represent a promising strategy of drug delivery with the controlled release [27]. The effectiveness of magnetic delivery systems includes the field strength, gradient, magnetic and physicochemical properties of the NPs [28]. Moreover, the main challenge of bare MNPs is to avoid their agglomeration due to their van der Waals and magnetic dipole–dipole attraction forces [13,29]. Considering their hydrophobic surface and rapid clearance from the blood through the reticuloendothelial system (RES), they are not suitable for drug delivery systems [22]. Therefore, to overcome this inconvenience, it was necessary to coat the magnetite NPs to reduce the aggregation tendency, protect their surface from oxidation, and make the particles biocompatible and stable [30].

Various polymers have been used for drug delivery, with polyethylene glycol (PEG) being the gold standard and the most commonly used polymer [31]. PEG is approved by the Food and Drug Administration (FDA) for internal use in humans and its products have been on the market for 20 years [23,31]. Since 1994, Gref and co-workers have reported on the PEG coating and demonstrated that the naked particles were removed from the liver only 5 min after injection [31]. PEG coating is extensively used in the preparation of NPs for biomedical applications due to its many advantages, such as stability in physiological media, prolonged half-life in the body, biocompatibility, and water solubility. Moreover, PEG coating prevents or reduces aggregation and confers better physical stability to drugs through steric and hydric repulsion [19,31].

In this study, we synthesized MNPs, which are known to have excellent biocompatibility and negligible toxicity [26], allowing their application in therapy. Prepared by the solvothermal method and stabilized by the widely used PEG, MNPs possess unique properties, such as colloidal stability, dispersibility, high porosity, high loading capacity, and specific morphological, thermal, and magnetic properties, especially superparamagnetism, which enable them to host the unstable or water-insoluble drugs and to direct and localize the drugs to the specific site in the tissue. The synthesized MNPs were fully characterized in terms of structural, morphological and magnetic properties. In addition, the kinetics of quercetin from the MNPs were controlled in vitro by simply varying stationary and alternating magnetic fields, resulting in fine-tuned manipulation of the released quercetin as a model drug. It should be noted that in this study cytotoxicity has been considered, but it was not a priority at this stage of our research. Regarding the measurement and result in the cytotoxicity literature of MNPs, we quite rightly expected at least the same cytotoxicity as the results obtained by Barreto et al. [3], Hua et al. [32] and Luo et al. [33]. It was shown that the system developed provides prolonged quercetin release, which is an important characteristic of targeted drug delivery systems. The enhanced quercetin release at the lower stationary magnetic field and higher frequency alternating magnetic field, together with the synergism of chemical and physical, i.e., superparamagnetic properties of MNPs, demonstrate the great potential of MNPs as a promising targeted drug delivery system with high potential for their, both therapeutic and diagnostic activity

## 2. Materials and Methods

### 2.1. Chemicals

Iron (III) chloride hexahydrate (97%) was purchased from Alfa Easar (Ottawa, ON, Canada). Ammonium acetate and polyethylene glycol (PEG, Mw = 4000 Da) were obtained from Sigma Aldrich (St. Louis, MO, USA). Ethylene glycol and ethanol (96%) were purchased from Lach-ner (Neratovice, Czech Republic). Compressed nitrogen was purchased from Messer (Bad Soden am Taunos, Germany). Silicon oil was received from Acros organics (Waltham, MA, USA). Phosphate buffered saline (PBS) buffer (PBS tablets, pH 7.4, *I*_c_ = 150 × 10^−3^ mol dm^−3^) were purchased from Sigma Aldrich (St. Louis, MO, USA). Deionized water Millipore mili Q-H2O was used to prepare the PBS medium. Quercetin (≥99%) was supplied by Lach-ner (Neratovice, Czech Republic). A molecular weight cut-off dialysis bag (MWCO, 8Kd) was purchased from Thermo Fisher Scientific (Waltham, MA, USA).

### 2.2. Synthesis of Bare and PEG Coated Magnetite MNPs

**Figure 1:**
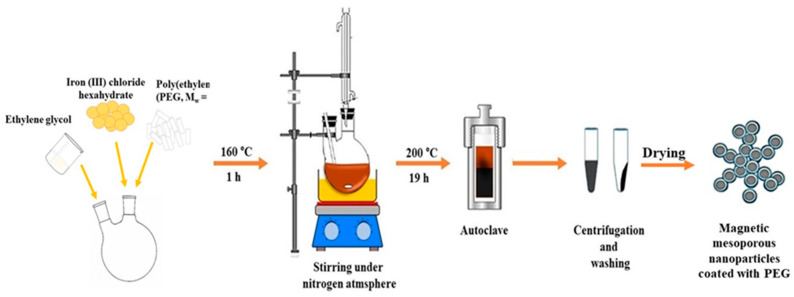
Schematic illustration of mesoporous MNPs preparation using solvothermal method.

The modified solvothermal reaction was used for the preparation of mesoporous magnetic nanoparticles [33,34]. Briefly, 1.35 g FeCl_3_ × 6H_2_O, 3.85 g of CH_3_COONH_4_, 1.0 g PEG (M = 4 kD) and 70 mL of ethylene glycol were added to a 250 mL two-necked flask equipped with a magnetic rod. The mixture was stirred vigorously for 1 h at 160 °C with a Heidolph MR Hei-Standard mixer (Schwabach, Germany). The chemical reaction was under the protection of an inert nitrogen atmosphere to form a homogeneous brownish solution (see Figure 1). After an hour, the system was stopped and cooled to room temperature. The mixture was transferred into a Teflon-coated stainless-steel autoclave (BHL 800 Berghof, Eningen, Germany) which is connected to a temperature controller. The autoclave was heated to 200 °C and maintained for 19 h and, afterwards, it was cooled to 50 °C. To remove the solvent, MNPs were centrifugated (Universal 320 Hettich Zentrifugen, Tuttlingen, Germany) at 8000 rpm for 10 min. After separation, MNPs were washed several times with ethanol and dispersed with a shaker (IKA Shaker Vortex 1, Staufen, Germany) between each wash. Finally, MNPs were left to dry in a desiccator for further characterization. For comparison purpose and characterization of NPs, bare magnetite MNPs were resynthesized each time using the same procedure.

### 2.3. Characterization of Synthesized Magnetite MNPs

The structural characteristics were determined by X-ray powder diffraction using Philips MPD 1880 diffractometer (Brooklyn, NY, USA) with monochromatic CuKα radiation (*λ* = 0.154 nm) at room temperature. The structural features of prepared samples were recorded at 2*θ* angles in the range of 10°–70° with a step of 0.02° and fixed time of 10 s per step. X-ray diffraction in polycrystalline is used to confirm crystalline size and structure of bare and coated magnetite MNPs obtained by solvothermal method. Field emission scanning electron microscope, JEOL JSM-7000F (Tokyo, Japan), was used to determine the morphology, particle size distribution and surface texture of bare and PEG-coated magnetite MNPs. FE-SEM was linked to the EDS/INCA 350 (energy dispersive X-ray analyzer) manufactured by Oxford Instruments Ltd., London, UK. The morphology of the MNPs has been further investigated using atomic force microscopy (AFM). The samples for AFM imaging were prepared by deposition of a magnetite MNPs suspension on the mica substrate. The MNPs are rinsed three times with 50 μL of MiliQ water to remove all residual impurities. AFM images were obtained by scanning the magnetite MNPs on the mica surface in the air using MultiMode Scanning Probe Microscope with Nanoscope IIIa controller (Bruker, Billerica, MA, USA) with SJV-JV-130 V (“J” scanner with vertical engagement); Vertical engagement (JV) 125 μm scanner (Bruker Instruments, Inc., Bruker, Billerica, MA, USA). Tapping mode was performed for imaging using silicon tip (R-TESPA, Bruker, Nom. Freq. 300 kHz, Nom. spring constant of 40 N/m) at 25.0 °C, allowing thermal equilibration before each imaging. AFM images were collected using random spot surface sampling (at least two areas per sample) for each analysed sample. All the images were processed by first-order flattening only and analysed using the NanoScope Analysis software (Version 5.31r1). Morphology analysis was investigated by Transmission Electron Microscope (TEM), Zeiss EM10 (Oberkochen, Germany), operated at 100 kV. In this propose, MNPs coated with PEG were dispersed in Milli-Q H_2_O and bare MNPs were dispersed in ethanol, ultrasonicated and placed on carbon coated copper grids. After air drying, samples were photographed by transmission electron microscopy. Samples were analysed with ImageJ and 50 particles were counted for each image.

The MNPs mesoporosity determination has been performed using nitrogen adsorption–desorption measurements on an ASAP2020 (Micromeritics, Norcross, GA, USA) accelerated surface area analyzer at 77 K. Before measuring, the samples were degassed at reduced pressure andat 120 °C for at least 6 h. All measurements have been made in duplicate. The specific surface area, the pore volume and the pore size are determined using Brunauer–Emmett–Teller (BET) analysis. To confirm the superparamagnetic property of the synthesized MNPs, magnetization measurements were performed. Magnetization of the powder samples of MNPs was measured using a commercial Quantum Design MPMS-5 SQUID magnetometer (San Diego, CA, USA). The powder samples were placed into a small ampoule whose diamagnetic contribution was properly subtracted. In addition, the field dependence of the magnetization (M(H)), including magnetic hysteresis loops, was measured at 300 K under fields up to 10 kOe.

### 2.4. The Determination of Loaded Quercetin into Magnetic Mesoporous MNPs

The quercetin loading efficiency into mesoporous MNPs has been confirmed using Brunauer–Emmett–Teller (BET) analysis. The difference in the specific surface area, the pore volume and the pore size before and after immersing the MNPs into the quercetin solution confirmed the loading of quercetin into MNPs. FTIR spectra were obtained by Alpha-T FTIR Spectrometer (Bruker, Billerica, MA, USA). All spectra were recorded between 4000 and 350 cm^−1^ at a nominal resolution of 4 cm^−1^ at 25 °C, and the total number of recordings was 16. Dried samples were mixed with KBr powder and then they were pressed to produce pellets. TG analysis data were carried out on a TG/DTA simultaneous analyser DTG-60H with a 10 °C/min heating rate under a nitrogen atmosphere. The measurements were recorded in a range of room temperature up to 1200 °C.

UV/VIS spectrophotometer was used to study the quercetin loading efficiency of the synthesized MNPs. The loading efficiency was calculated by measuring the absorbance of the supernatant with the WTW photoLab^®^ 7600 UV-VIS Spectrophotometer (Xylem, Rye Brook, NY, USA) at 374 nm. Measurements were performed in a rectangular quartz cuvette with a 10 mm optical path length and covers at 25 °C.

A total of 500 mg of quercetin was dissolved in 100 mL of ethanol and suspended in an ultrasonic bath (Bandelin Sonorex Super RK 100 H, Berlin, Germany) for 1 h at room temperature. The 25 mL aliquot of quercetin solution and 100 mg of coated MNPs were transferred into a 50 mL Falcon conical centrifuge tube. The mixture was mechanically stirred at a thermocontrol shaker (Barnstead Lab-line 4450 e-class) for 24 h at 200 rpm and 25 °C. Afterwards, the quercetin-loaded MNPs were separated from unloaded quercetin by centrifugation (Universal 320 Hettich Zentrifugen, Tuttlingen, Germany) at 8000 rpm for 15 min. Compared with quercetin concentration supernatant before adding the synthesized MNPs, the concentration loss was determined using a calibration curve in pure EtOH. The coefficient of determination was 0.9978, and the determined molar absorption coefficient of quercetin at temperature 298 K and 374 nm is 19,131 mol^−1^ cm^−1^ dm^3^. The loading efficiency (*LE*) was calculated by measuring the absorbance of the supernatant with the Photolab 7600 UV-VIS spectrophotometer (Xylem, New York, NY, USA) at *λ* = 374 nm. The absorbance (*λ* = 374 nm) was collected and converted to concentration by using the equation from the calibration curve. Therefore, the drug loading efficiency was calculated as the following equation:

(1) $$LE=\frac{m_{\mathrm{embedded}}}{m_{\mathrm{NP}}}\;\times \;100\%$$

where *m*_embedded_ represent the mass of quercetin incorporated in nanoparticles, and *m*_NP_ is the total mass of MNPs which is used for loading.

#### Size Distribution of Magnetic MNPs Using Dynamic and Electrophoretic Light Scattering

Hydrodynamic diameter (*d*_H_) and zeta potential (*ζ*) of suspended MNPs were measured by photon correlation spectrophotometer, Zetasizer Nano ZS (Malvern, UK) with green laser (*λ* = 532 nm) using the M3-PALS technique. All measurements were conducted at 25 °C in PBS (pH = 7.4) buffer. The hydrodynamic diameter was determined from the peak maximum of the volume size function. The zeta potential (*ζ*) was calculated from the electrophoretic mobility using a Smoluchowski approximation (*f*(κa) = 1.5). The *d*_H_ values were obtained as an average value of 10 measurements, while the zeta potential values were reported as an average of 3 independent measurements. The results were collected by the Zetasizer software 6.32 (Malvern Instruments, Malvern, UK).

### 2.5. Release Study of Quercetin under Stationary and Alternating Magnetic Fields

**Figure 2:**
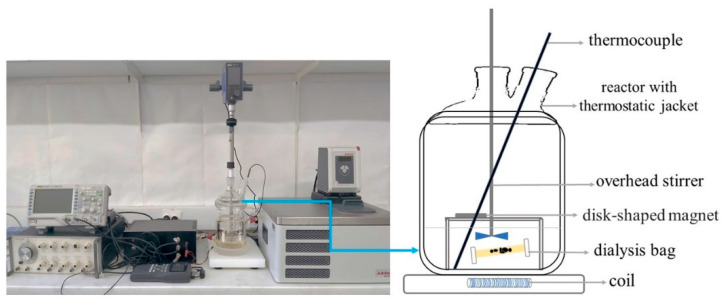
Experimental setup of the release kinetics and schematic illustration of a reactor.

The apparatus setup scheme shown in Figure 2 enables the fine-tuning of the quercetin-release kinetic profile under applied combined stationary and alternating magnetic fields. The magnetic field enforced in the experiment is a combination of stationary and alternating magnetic fields. The position of the permanent magnet fixed and stable towards the fixed magnetic coil placed within the reactor ensures the permanent and alternating magnetic fields perpendicular to each other [35]. Without going into more detail, we showed in previous work [35] that, by applying external magnetic fields, MNPs behave as Brownian particles with quasi-periodic movement that enables loaded drug molecules to became enhanced released from the MNPs. A magnetic field is an effective stimulus with deep penetration capacity. The high-frequency alternating magnetic field (HF-AMF), from 50 to 400 kHz, and low-frequency alternating magnetic field (LF-AMF), from 0.1 to 5 kHz, have been employed to trigger the release of drugs from nanocarriers [36]. In our previous work, we employed the LF-AMF to induce the flavonoid release from the magnetic aggregates [35]. To get more insights into the effect of the combination of applied permanent and AMF to drug release, we designed sophisticated HF-AMF instrument to induce increased drug release at HF-AMF having in mind Brezovitch criterion [37]. He proposed a safety limit where the product of magnetic field and amplitude frequency (H_0_×f) should not exceed 4.85 × 10^8^ A m^−1^ s^−1^ to avoid any harmful effect on the organism. In our study, the product amounts 4 mA m^−1^ s^−1^, 1 mA m^−1^ s^−1^ and 0.3 mA m^−1^ for 100 kHz, 50 kHz and 10 kHz, respectively, indicating that we chose good frequencies and applied magnetic fields for any possible safe application to patients. In consideration of the design of our experiment, where there were relatively high frequencies of alternating magnetic fields (from 10 to 100 kHz), it was expected that the release of quercetin would be significantly enhanced by the influence of higher frequency.

The drug release was calculated by measuring the absorbance of quercetin released in the supernatant at *λ* = 330 nm by the UV-Vis spectrophotometer. The releasing kinetics of quercetin from magnetite MNPs was investigated at three temperatures (25 °C, 30 °C and 37 °C) in the mixture PBS/EtOH (vol. 50/50) in which the solubility of quercetin totals to 5.66 mg/mL, 5.83 mg/mL and 6.02 mg/mL at 25 °C, 30 °C and 37 °C, respectively [38]. For the use of NPs as a drug delivery system, a physiological temperature of 37 ° C is of crucial importance. However, before the application of MNPs, they must be adequately stored. There are studies on the storage of MNPs at low temperatures, and the influence of low temperatures on the magnetic properties of MNPs [39]. Although the temperature effects on the magnetic properties of magnetite are very weak, MNPs contain a coating of organic material, in our case PEG 4000. Since PEG 4000 has crystallization temperatures at around 32 °C [40], which means that by changing the temperature of the MNPs suspension from the storage temperature to the ambient temperature, and further to the physiological temperature of 37 °C, the surface properties of MNPs and the relaxation kinetics could be changed. For this reason, measurements were made not just at 37 °C, but also at 25 and 30 °C to see how much the temperature change affects the drug-release kinetics. In addition, similar studies of the mechanism of release kinetics from NPs at different temperatures (10, 22 and 37 °C) have been conducted, for example, in study by Gronczewska [41], in that drug release and matrix degradation of polymer microspheres with different glass transition temperatures were investigated at various temperatures in order to clarify the effect of temperature on mechanisms of drug release. Then, 100 mg of quercetin-loaded MNPs were transferred in a dialysis bag (MWCO 8 kD, Thermo Fisher Scientific (Waltham, MA, USA)), after which 1 mL of PBS/EtOH (Vol. % = 50:50) medium was added and closed with dialysis bag clip holders. The dialysis bag was placed in a glass cylindrical reactor with a thermostatic jacket and flange size LF 100 containing 150 mL of PBS/EtOH mixture. The overhead stirrer (Ministar 20 control, IKA-Werke GmbH&Co (Staufen, Germany)) is going through the centre neck flat flange lid. The stirrer was set to 200 rpm. At selected time intervals, 1 mL of supernatant was replaced with fresh PBS/EtOH mixture through one angled side neck flat flange lid. The reactor is connected to a refrigerated–heating circulator (Corio CD-201F Julabo GmbH (Seelbach, Germany)) to control the appropriate temperature. The controlled release of quercetin was tested using appropriate external alternating (10 kHz, 50 kHz and 100 kHz) and stationary magnetic fields (*B* = 7.9 mT and 11.0 mT) at controlled electric current (*I* = 100 mA). An external alternating magnetic field was achieved with a function generator (Wavetek 164 30 MhZ (San Diego, CA, USA) connected to the coil (N = 270, *l* = 4 cm). Experiments were performed using the magnetic field system set up from permanent disk-shaped magnets (rare earth) and solenoid with permalloy core connected to signal generator alternating 100 mA current. A reactor with the sample was placed among the two magnetic fields. Defining the *O*_xy_ plane as the surface of the liquid, the permanent field was along the *O*_z_ axis and the alternating field along the *O*_x_ axis. Weak fields were applied in all experiments: the strength of the static permanent magnetic fields at the appropriate distance between the membrane dialysis bag and the permanent magnet was *B* = 7.9 mT and 11.0 mT. They were placed on a stand inside the reactor and used as sources of the permanent magnetic field. The function generator is connected to the oscilloscope (DS1000Z, Rigol Technologies (Beijing, China), which allowed the observation of the sinus waveform signal. The release kinetics of quercetin from magnetite MNPs was quantified by UV/VIS spectrophotometry (Photolab 7600 UV/VIS spectrophotometer Xylem (New York, NY, USA)). The linearity of the calibration was found to be valid from 1 × 10^−6^ mol dm^−3^ to 1 × 10 ^−4^ mol dm^−3^ with correlation coefficients for quercetin all approaching 1.00. All release kinetics experiments have been performed in duplicate.

## 3. Results and Discussion

### 3.1. Characterization of Synthesized Magnetite MNPs

**Figure 3:**
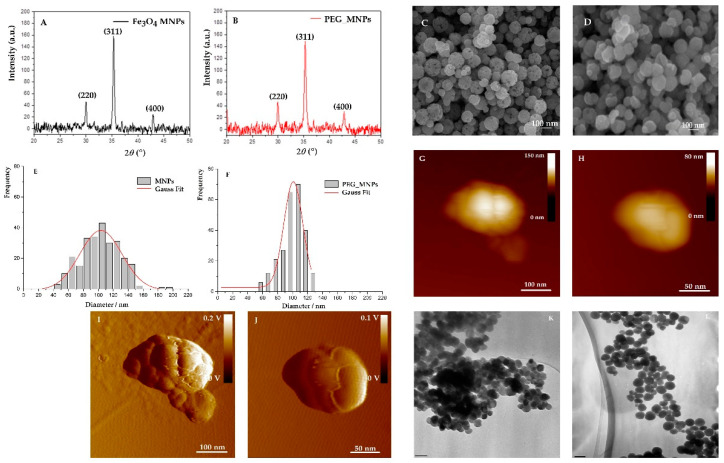
XRD pattern of (**A**) mesoporous bare MNPs Fe3O4; (**B**) PEG-coated MNPs; SEM images of (**C**) bare Fe3O4 MNPs showing the rough rounded cluster with extensive open porosity ranging from 30 to 200 nm in diameter; (**D**) SEM image of the surface of PEG-coated Fe3O4 MNPs ranging in di-ameter from 50–130 nm; Histogram of bare MNPs Fe3O4 (**E**) and PEG-coated Fe3O4 MNPs (**F**); (2D height topographic AFM image of single (**E**) bare magnetite Fe3O4 MNPs (**G**) and (**F**) PEG-coated Fe3O4 MNPs (**H**) showing cluster structure details; 2D-amplitude AFM image of single (**G**) bare magnetite (**I**) and (**H**) PEG-coated MNPs (**J**) showing subcluster structures in size range from 10 to 30 nm; TEM images of (**K**) bare Fe3O4 MNPSs; (**L**) TEM image of the surface of PEG-coated Fe3O4 MNPs.

The X-ray powder diffraction patterns of the synthesized bare and PEG-coated MNPs are presented in Figure 3A,B, respectively. Characteristic peaks exhibited in the XRPD pattern are well-matched with the magnetite diffraction peaks and confirm the cubic inverse spinel structure of MNPs. The sharp diffraction of three characteristic peaks (220), (311) and (400) also indicate the spinel structure of magnetite [42]. The formation of pure cubic magnetite is confirmed by the value of the calculated lattice parameter “*a*” which has been determined to be 8.389 Å [43]. Using Scherrer’s equation, the average crystallite size of bare and PEG-coated MNPs were calculated to be 25 nm and 19 nm, respectively. In addition, the decreased average crystallite size, another piece of evidence suggesting that PEG decreases the crystallinity of MNPs at a lower intensity and with broader diffraction peaks than PEG-coated MNPs.

The size and morphology of bare and PEG-coated mesoporous MNPs were observed by field-emission scanning electron microscope (FE-SEM), atomic force microscopy (AFM) and transmission electron microscopy (TEM), as shown in Figure 3A,B,G–J,K–L). Both bare and PEG-coated MNPs maintain a uniform spherical shape, some of them agglomerated due to magneto–dipole interactions between MNPs. While bare MNPs showed cluster structure with a very rough surface, the process of PEG coating revealed the flattened surface of mesoporous MNPs (Figure 3I,J). These findings have been confirmed by AFM imaging where the roughness of the MNPs surface has been increased by PEG coating, for almost 100%, from 5.58 ± 1.06 nm to 10.9 ± 2.1 nm, confirming the effective coverage of the bare MNPs by PEG [43]. The less agglomerated texture of the PEG-coated MNPs can be related to the effect of the PEG layer during the synthesis of MNPs. In addition, a size histogram of bare mesoporous MNPs obtained using SEM micrographs shows a broader size distribution than PEG-coated MNPs (*d*_ave_ = 103.4 ± 0.7 nm and 101.0 ± 0.9 nm for bare and PEG-coated MNPs, respectively), indicating that polymer decreased the magnetic interaction among the particles and prevent their agglomeration. The cluster structure of mesoporous MNPs has been confirmed using AFM. The average size of bare MNPs and PEG-coated MNPs corresponds to results obtained by SEM, it was even 10% larger due to the convolution effect. However, in both 2D height images, it is shown that the cluster structure MNPs consists of smaller substructures, in the range of 15 to 35 nm, which roughly correspond to the dimension of the crystallite size obtained by X-ray powder diffraction. Hence, AFM revealed that only several nanometers increase in roughness is observed when PEG 4 kD is used, suggesting that the PEG layer is largely twisted the magnetite surface, rather than stretch out linearly [44]. Furthermore, this PEG layer should decrease the magnetic interactions among the MNPs and prevents their agglomeration. The mean diameter of bare MNPs obtained by TEM is larger (143 ± 30 nm) than obtained by SEM (103.4 ± 0.7 nm), indicating a higher degree of polydispersity. However, the mean diameter of PEG-coated MNPs (96 ± 10 nm) corresponds the value obtained by SEM (101.0 ± 0.9 nm). Increasing the size of the PEG-coated MNPs can be attributed to the successful coating of PEG. As shown in Figure 3K,L, both MNPs maintain a typical spherical shape.

**Figure 4:**
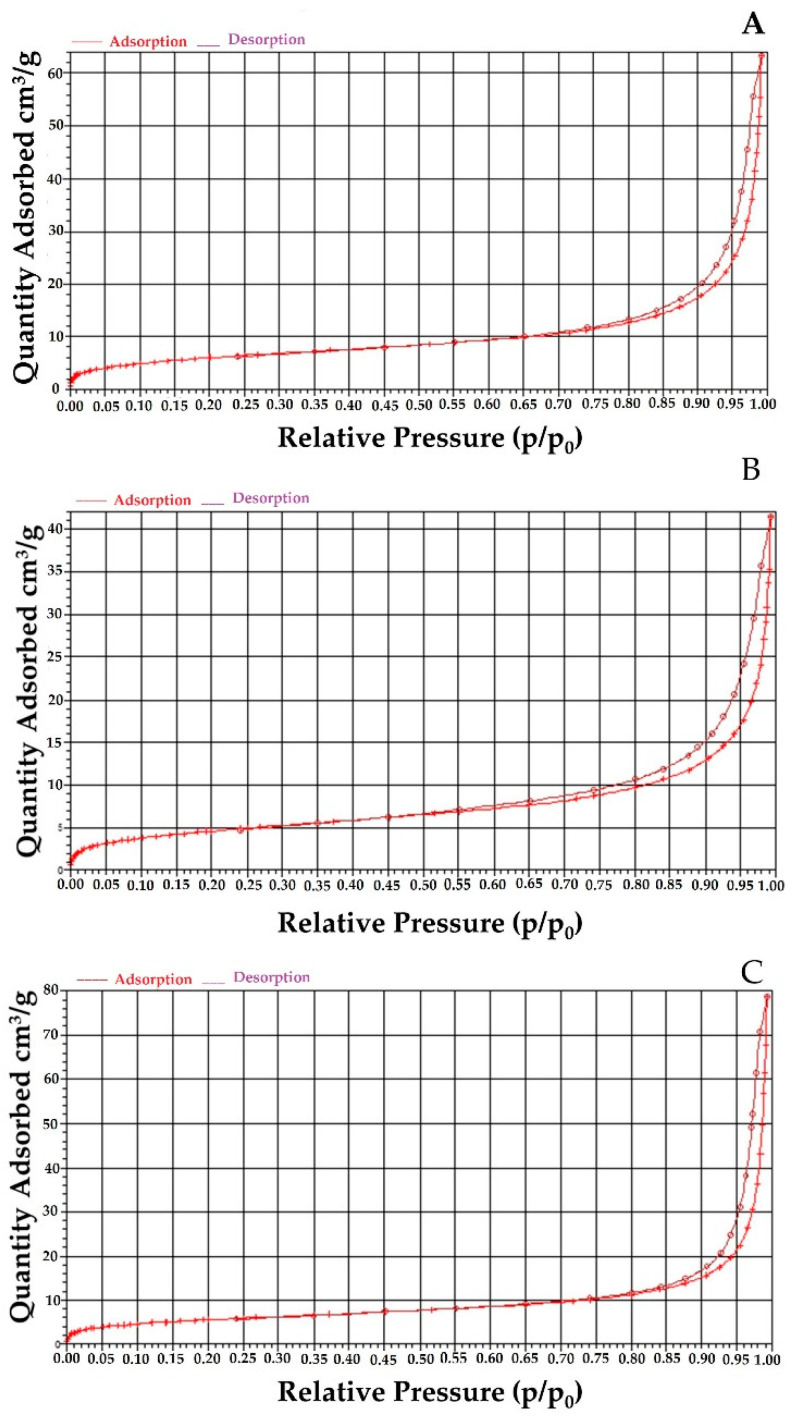
N_2_ absorption-desorption isotherm for (**A**) bare MNPs, (**B**) PEG-coated MNPs and (**C**) quercetin-loaded PEG_MNPs.

Nitrogen adsorption–desorption isotherms of bare MNPs confirmed their mesoporosity (see Figure 4). The surface area, pore size and total pore volume were calculated to be 20.5 m^2^g^−1^, 17.7 nm and 0. 09 cm^3^ g^−1^, respectively, strongly supporting the fact that the bare MNPs have mesoporous structure. The PEG coating of bare MNPs led to a slight decrease in surface area (19.3 m^2^ g^−1^) but an increase in pore size and total pore volume (24.6 nm^1^ and 0.11 cm^3^ g^−1^, respectively).

**Figure 5:**
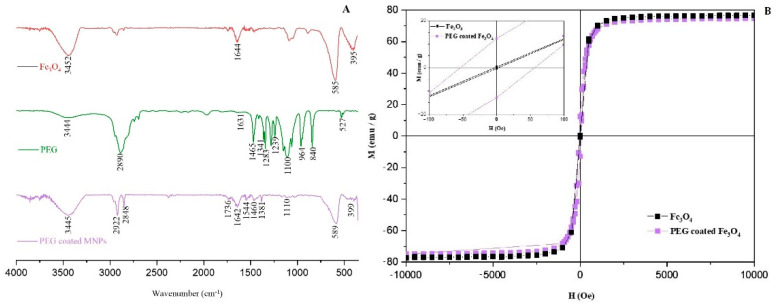
(**A**) FTIR spectra of MNPs, PEG and PEG-coated MNPs (**B**) Magnetic behavior of MNPs. The hysteresis loop shows a slight decrease in the magnetization behavior after a thin PEG layer coating. The magnetic hysteresis loops of mesoporous MNPs at 300 K. Inset within Figure 5 (**B**) shows the magnetic coercivity *H*_c_ = 53.3 Oe.

FTIR spectrum (Figure 5A) of bare magnetite MNPs shows bands at 585 and 395 ${\mathrm{cm}}^{-1}$ corresponding to the symmetric stretching vibration mode of the Fe-O bond. The absorption maxima at 3452 ${\mathrm{cm}}^{-1}$ and 1644 ${\mathrm{cm}}^{-1}$ suggest the presence of O-H linkages. In the pure PEG, the bands at 1341 and 1100 ${\mathrm{cm}}^{-1}$ belong to the C-O-C ether bond asymmetric and symmetric stretching vibrations. The band at 2890 cm^−1^ is attributed to -CH_2_- stretching vibration in PEG. In addition, absorption bands at 1283 and 1465 cm^−1^ attributed to the vibration of–CH_2_ and at 964 cm^−1^ corresponds to the CH out-of-plane vibration [18]. The hydroxyl groups were also confirmed at 3444 and 1631 cm^−1^. The presence of characteristic FTIR bands of PEG in PEG-coated MNPs spectrum confirmed the successful coating of PEG on the surface of magnetite MNPs. The PEG-coated MNPs spectrum shows a strong C-O-C ether stretch at 1110 and 1381 cm^−1^ [44]. In addition, the O-H linkages at the 1642 and 3445 cm^−1^ bands exhibited enhanced intensity which also indicates that PEG modified the surface of MNPs. The transmittance bands at 589 and 399 cm^−1^ confirm the symmetric stretching mode of the Fe-O bond. The results indicate that PEG is successfully functionalizing the surface of MNPs.

In order to confirm the superparamagnetic properties of synthesized bare and PEG-coated MNPs, measurements of the magnetization curve have been performed. The S-shaped hysteresis loops are a typical feature of superparamagnetic MNPs and obtained result is very similar to our previous work (77 emu g^−1^) on Fe_3_O_4_ MNPs [35]. The magnetization curve clearly shows that magnetization depends on the applied magnetic field, but not on the sign of the applied field. Magnetic characterization at 300 K indicates that the bare MNPs have saturation magnetization at the maximum field of 5 kOe value of 76.71 emu g^−1^, which is lower than obtained value for the bulk Fe_3_O_4_ (*M*_s_ = 92 emu g^−1^) [42] or *M*s = 96.43 emu g^−1^ [19].

The observed decrease in the saturation magnetization could be explained either by the use of PEG for the surface modification process [43] that causes the softening of the magnetization or by the difference in particle size [45]. In addition, the saturation magnetization of PEG-coated MNPs at the maximum field of 5 kOe is 74.75 emu g^−1^, somewhat lower than bare MNPs (Figure 5B). However, the magnetization measurement of synthesized MNPs confirmed their superparamagnetic properties, thus confirming their further application in release studies under applied magnetic fields.

### 3.2. Loading of Flavonoid into Magnetite MNPs

**Figure 6:**
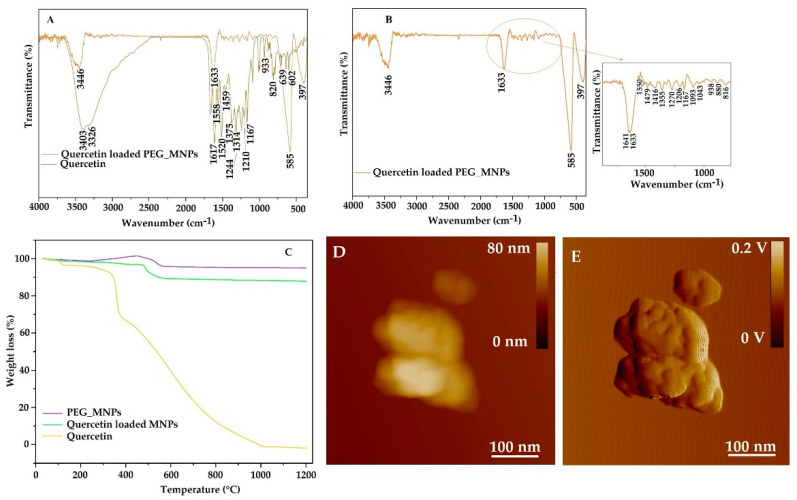
FTIR spectra of (**A**) quercetin and quercetin-loaded MNPs; (**B**) zoomed spectrum of quercetin-loaded MNPs in the region of quercetin reach bands; (**C**) TGA curves of PEG-coated MNPs, quercetin and quercetin-loaded MNPs. Morphology of quercetin-loaded MNPs on the 2D height AFM image (**D**) and 2D-amplitude AFM image (**E**).

The FTIR spectrum (Figure 6A,B) of quercetin detected OH group stretching at 3403 cm^−1^ and 3328 cm^−1^. The band at 1664 cm^−1^ corresponds to the C = O aryl ketonic stretching and the bands at 1617 cm^−1^, 1558 cm^−1^ and 1520 cm^−1^ correspond to C = C aromatic ring stretching. OH bending of the phenol functional group at 1375 cm^−1^ and 1314 cm^−1^ belongs to the in-plane bending of C–H in aromatic hydrocarbon. Bands at 933 cm^−1^, 820 cm^−1^, 639 cm^−1^ and 602 cm^−1^ correspond to aromatic C–H out-of-plane bendings. The C–O stretching in the aryl ether ring and the C–O stretching in phenol corresponds to 1244 cm^−1^ and 1210 cm^−1^ transmittance maxima. The band at 1167 cm^−1^ is attributed to the C–CO–C stretch and bending mode in ketone, respectively. The FTIR spectra of quercetin-loaded MNPs show the broadening of the OH band at 3446 cm^−1^, which confirms the entrapment of quercetin in MNPs [46]. The interval from 1560 to 816 cm^−1^ matches very well with pure quercetin peaks and indicates successful loading [46].

The loading of quercetin has been also confirmed using nitrogen adsorption–desorption isotherms of quercetin-loaded PEG MNPs. In comparison to PEG covered MNPs, the surface area has been decreased by almost 19% to 15.7 m^2^ g^−1^, pore size decreased to 14.2 nm for 42.4%, while total pore volume amounting 0.06 cm^3^ g^−1^, decreased for 49% to 0.06 cm^3^ g^−1^, strongly supporting the fact that the quercetin has been effectively loaded into MNPs.

The thermogravimetric study was performed to confirm the quercetin loading in MNPs. Figure 6C shows comparative weight loss for quercetin and quercetin-loaded MNPs. A thermogravimetric study of quercetin reveals that the compound undergoes a three-stage thermal decomposition. The first stage of mass loss begins at 30 °C and continues up to 133 °C. A mass loss of 3.52% is attributed to dehydration or loss of water molecules on the surface of quercetin. In the temperature range 133 °C to 385 °C, the compound experiences a weight loss of 28.5% due to the melting of quercetin. The final thermal decomposition is observed in the temperature range of 385 °C to 1110 °C, and the weight loss of quercetin is 67% [47]. In the case of quercetin-loaded MNPs, the weight loss in the temperature range from 30 °C to 1200 °C is about 12.2%, which is attributed to the decomposition of organic compounds from MNPs. This data results in the great thermal stability of quercetin when it has been encapsulated in MNPs. In the case of PEG-coated MNPs, there is an increase in the weight gain resulting from the burning of the PEG and oxygenation of Fe_3_O_4_ starting at 270 °C under the continuous flow of oxygen at high temperatures and finishing at 450 °C [48]. However, the weight loss of PEG from the PEG-coated MNPs amounts to 4%. It is assumed that the thermal decomposition of PEG occurs at both C-O and C-C bonds of the backbone chain [49]. The influence of the quercetin loading into magnetite MNPs on the size and morphology of the MNPs has been further investigated. 2D height AFM image (Figure 6D) and 2D-amplitude AFM image (Figure 6E) revealed that distinct subcluster structure containing MNPs has been retained irrespective of quercetin loading, with size grains from 20 to 50 nm in diameter. The roughness value after quercetin loading decreased from (10.9 ± 2.1) nm to (4.86 ± 1.1) nm additionally confirming the successful loading of quercetin to PEG loaded MNPs.

In addition, UV/VIS spectroscopy is used to study quercetin loading efficiency. Compared with quercetin concentration before adding the synthesized MNPs, the concentration loss was determined using a calibration curve in pure ethanol (EtOH). The coefficient of determination was 0.9978, and the determined molar absorption coefficient of quercetin at 298 K and 374 nm is 19,131 mol^−1^ dm^3^ cm^−1^. The loading efficiency (LE) was determined to be (20.2 ± 1.3%) calculated from 17 independent experiments. Our results suggest significant improvement of the loading efficiency of the quercetin compared with a loading efficiency of solid lipid NPs (13.20 ± 0.18%) [1]. However, the limited LE is due to the reduced specific loading site of quercetin induced by PEG coatings [50]. Despite this, the PEG –MNPs provide the capability to load antioxidant quercetin with low aqueous solubility which reflects the potential of the synthesized MNPs as drug delivery carriers.

### 3.3. Homogeneity and Stability of Synthesized Mesoporous MNPs

One of the essential features for successful drug delivery is stability and homogeneous dispersion of NPs in buffer. Zeta potential was used to determine the stability of the colloidal suspension of bare MNPs, PEG-coated MNPs and quercetin-loaded MNPs. It has been shown that PEG-stabilized NPs exhibit longer bloodstream circulation time and higher resistance to protein binding [44]. Due to its unique properties and its biocompatibility, PEG is selected as the stabilizing agent in this study. The surface charge of NPs has an important effect on the blood circulation time, the pharmacokinetics of NPs and the zeta potential above ±30 mV indicated to be relevant for stability of NPs in aqueous suspensions [12,51,52]. The zeta potential of MNPs (Table 1) was determined in phosphate-buffered solution (PBS). Bare MNPs exhibited a zeta potential of (−30.6 ± 0.7) mV, while PEG coating increased the absolute zeta potential value to (−35.1 ± 1.5) mV. Indeed, the higher negative zeta potential value indicated that PEG-coated MNPs possessed higher stability after PEG coating. The zeta potential of quercetin-loaded MNPs (−31.3 ± 0.8) mV decreased slightly in comparison to PEG-coated MNPs indicating quercetin adsorption on the PEG layer of MNPs. However, the observed change in zeta potential value did not decrease the stability of MNPs. Moreover, the successful loading of quercetin into PEG-coated MNPs has been confirmed also by electrophoretic measurements.

**Figure 7:**
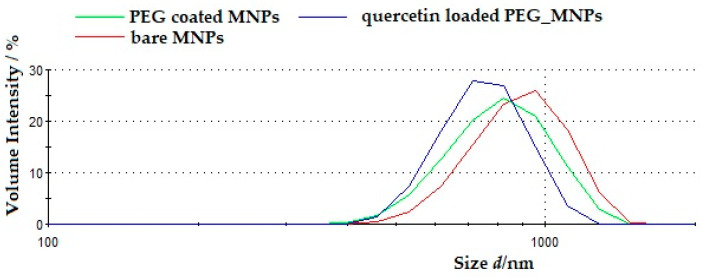
Volume size distributions of bare MNPs, PEG-coated MNPs and quercetin-loaded PEG_MNPs.

Furthermore, the hydrodynamic diameter is lower which could be attributed that PEG coating while quercetin loading provides better colloidal stability and reduces aggregation. The volume size distributions of all samples, i.e., bare MNPs, PEG-coated MNPs and quercetin-loaded MNPs were unimodal. In Table 1, it can be seen that the highest polydispersity index was observed to be 0.54 ± 0.1 for the bare synthesized MNPs suspension. This result is consistent with the results obtained from the size distribution of bare MNPs using SEM where the size distribution of bare MNPs was broader than the size distribution of the PEG-coated MNPs. The observed discrepancy between SEM and DLS data, particularly in the polydispersity index (PDI), can be explained by the fact that the SEM micrographs were taken in a dried state, whereas the DLS experiment was carried out in suspension. The PDI results reported in Table 1 also support the conclusion that PEG coatings, even presented in suspension, decreased the process of aggregation of bare MNPs and break down the cluster. The average hydrodynamic diameter of both, bare and PEG-coated MNPs also supports the above findings. In Figure 7, it is shown the volume size distributions of bare MNPs, PEG-coated MNPs and quercetin-loaded PEG_MNPs. The PDI of PEG-coated MNPs is lower than PDI of loaded MNPs (PDI = 0.47 ± 0.1 and 0.52 ± 0.07, respectively) indicating narrower size distribution of the PEG-coated MNPs than quercetin-loaded MNPs (see Figure 7). However, the cluster sizes of quercetin-loaded MNPs were within the range of (681 ± 73) nm, which is the smallest range of all analysed MNPs. Thus, the PEG coating on the surface of Fe_3_O_4_ decreased the size compared to the bare MNPs and indicates that the PEG coating prevents or reduces aggregation to some extent.

### 3.4. Release Study

Before we start to evaluate the quercetin-release kinetics from MNPs into PBS/EtOH (Vol. % 50:50), we want to emphasize that the results were obtained by applying external stationary and alternating magnetic fields. The underlying idea was to enable fine-tuned and controlled quercetin-release kinetics, which is certainly very important in a widespread, high-demand application of high-demand drug delivery via nanocarriers.

**Figure 8:**
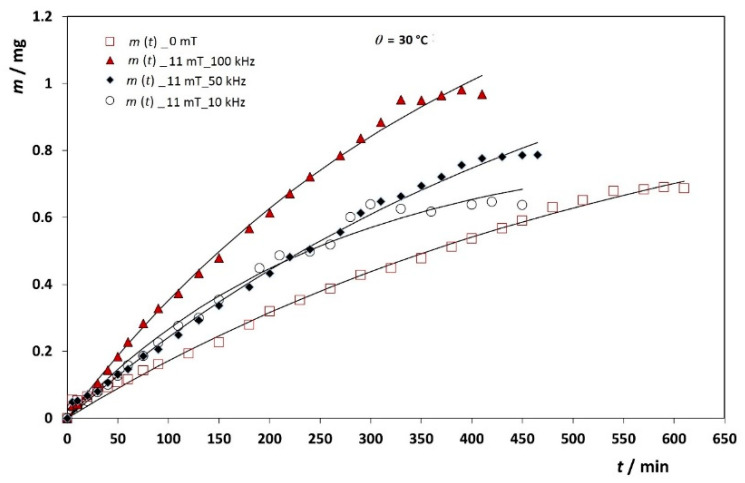
The representative cumulative release profiles for the quercetin from MNPs through dialysis membrane under the stationary magnetic field *B* = 11 mT and alternating field with frequencies 10 kHz, 50 kHz and 100 kHz at 30 °C.

The in vitro quercetin release profile from synthesized and carefully designed MNPs was studied in duration up to 8 h with a dialysis membrane and in the presence of external magnetic fields. The results are shown as the cumulative released mass of quercetin in Figure 8. Within 8 h, only up to 5% of quercetin has been released depending on the experimental conditions.

A similar sustained-release profile of quercetin from alginate NPs at pH 7.4 has been reported earlier [53], where only 10% of quercetin release was observed after 12 h, while after 9 days, only 50% of the quercetin got released. In another study, the quercetin release from the functionalized magnetite NPs was conducted in acidic and basic pH and cumulative release reached 3.7% after 6 h [14]. A similar prolonged release has also been found for quercetin release from polylactide NPs, which showed almost 60% quercetin released after 4 days [54]. The observed slow quercetin-release kinetics offers prolonged exposure to the drug and improves its efficiency compared with free drugs [55]. This is considered as an advantage because the burst release of drugs leads to a significant premature quantity of the drug that can result in toxicity [21]. For example, the quercetin at a concentration between 0 and 200 × 10^−6^ mol dm^−3^ could decrease antioxidant activity while quercetin at a concentration of (0.2–1) × 10^−6^ mol dm^−3^ possesses pro-oxidant activity [56]. In addition, quercetin release was affected by oxidative degradation process in PBS solution after continuously stirring for 6 h; this is yet another reason to employ nanocarriers for quercetin delivery [14,57,58]. Therefore, we prepared MNPs onto which quercetin easily adsorbs and has a prolonged stability and duration. The net result is quercetin release for a prolonged time. A first-order release profile of quercetin from Fe_3_O_4_-quercetin-copolymer NPs was revealed by Barreto et al. [3]. On the other hand, the release rate of quercetin from superparamagnetic magnetite NPs coated with chitosan, PEG and dextran was found to be of zero-order kinetics (linear with time) [41].

The assumptions in release kinetics experiments were as follows:

(i) The total amount of quercetin remained practically constant during the whole release experiment; (ii) both the quercetin solution within the membrane interior and in the membrane exterior were homogeneous due to the constant stirring and the fact that quercetin solution was never saturated; (iii) the thickness of the membrane provides the equal rate constants from the membrane interior to the membrane exterior, and vice versa; (iv) the volume within the membrane interior *V*_i_ and the volume exterior to the membrane *V*_o_ were constant during all performed release experiments. This is because *V*_i_ = 1 mL and *V*_o_ = 150 mL, *V*_i_ ˂ *V*_o_.

**Figure 9:**
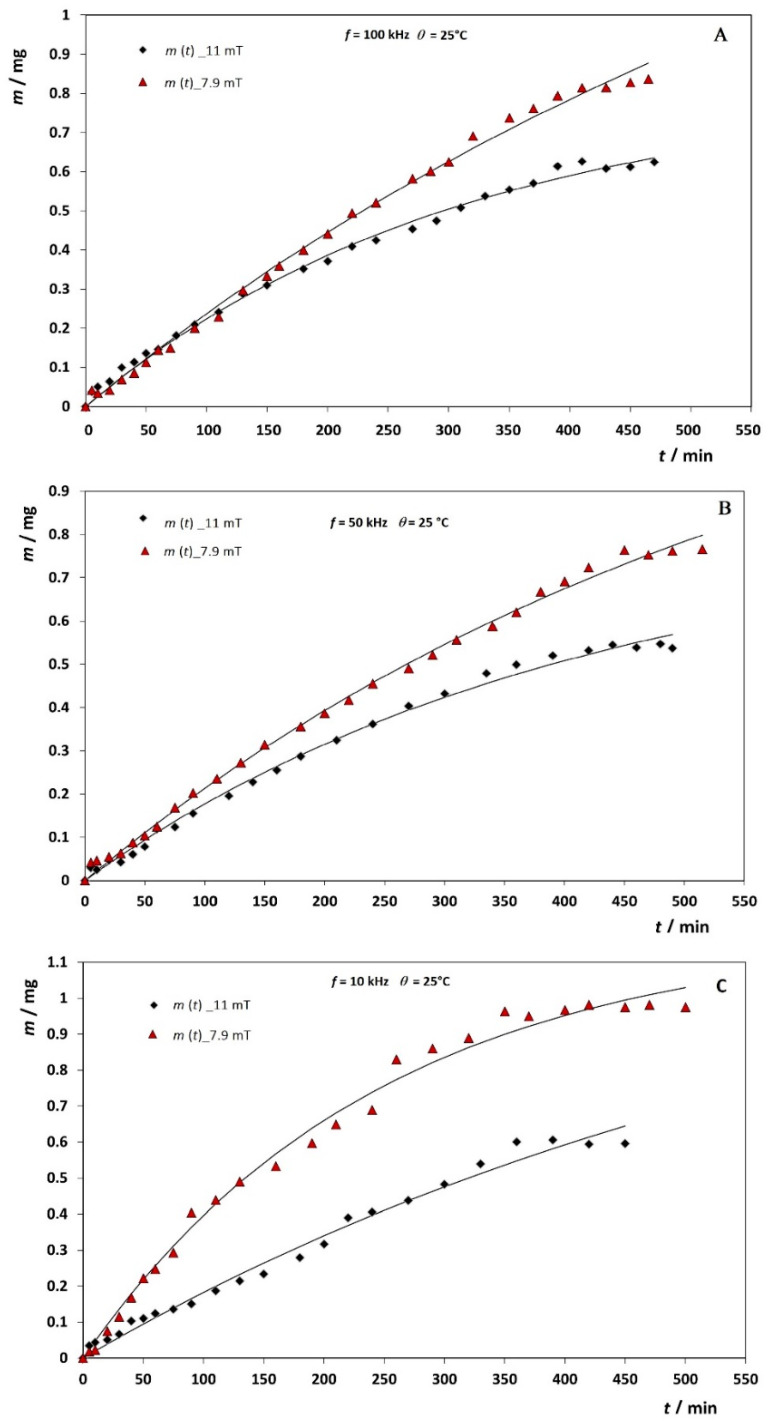
The representative cumulative release profiles for the quercetin from MNPs through dialysis membrane at 25 °C using two stationary magnetic fields and three different frequencies of alternating magnetic field (**A**) 100 kHz, (**B**) 50 kHz and (**C**) 10 kHz.

As a preliminary approach in working out the data, a simple model of single exponential decay was meant to be used in which the fraction of the released drug is Φ=1−exp(−kt), where *Φ* is a fraction of drug present in the outer volume *V*_0_ (other fractions are in the inner volume *V*_i_. *Φ*_i_, and in the membrane, *Φ*_m_) [59]. The coefficient *k* is related to the apparent kinetics and, as such, cannot provide information about the actual release rate from the nanocarriers into the interior volume *V*_i_. However, the problem with this simple formula is the equilibrium value $\Phi_{o}=1$ which is achieved when the elapsed time is sufficiently large (t→+∞). In our release kinetics experiments, it is invariably between 0.04 and 0.10. Thus, the dialysis bag with its content has to be considered as a source of drug molecules. It is not possible to know the total mass of the drug that is amenable to be released, but it can be estimated from the value of *m*_0_, which occurs in another simple model:

(2) $$m\left(t\right)=m_{0}\left(1-e^{-kt}\right),\;\frac{m}{m_{0}}=1-e^{-kt}$$

where *m*(*t*) is the released mass, not a fraction of, at time *t*, *m*_0_ is the total released quercetin mass from the dialysis bag after infinite time, and *k* is the rate coefficient of actual release kinetics of the dialysis bag membrane. The problem is that *m*_0_ is not experimentally well defined, i.e., it is only approximately constant across the series of experiments. Fitting the release experimental data obtained at various magnetic fields and at three different temperatures using this simple model (Table 2 and Figure 8 and Figure 9) resulted as expected in a fairly narrow interval of *m*_0_ values with average *m*_0_ = (1.48 ± 0.34) mg.

Each experiment was done in duplicate. This was fully justified to avoid averaging the measurement results and use the mean values of the two fitting procedures because the product, *kt*, was always rather small (Table 2). This was the case because, if $m_{1}=m_{0,1}\left(1-e^{-k_{1}t}\right)$, $m_{2}=m_{0,2}\left(1-e^{-k_{2}t}\right)$ and $m=m_{0}\left(1-e^{-kt}\right)$ where $m=1/2\left(m_{1}+m_{2}\right)$ and $m_{0}=1/2\left(m_{0.1}+m_{0.2}\right),$ the following is obtained:

(3) $$e^{-kt}=\frac{m_{0.1}}{m_{0.1}+m_{0.2}}e^{-k_{1}t}+\frac{m_{0.2}}{m_{0.1}+m_{0.2}}e^{-k_{2}t}$$

Since the product *kt* is always very small, it turns out that a simple formula,

(4) $$k=\frac{m_{0.1}}{m_{0.1}+m_{0.2}}k_{1}+\frac{m_{0.2}}{m_{0.1}+m_{0.2}}k_{2}$$

can be used. Furthermore, it is very often *m*_0,1_≈ *m*_0,2_ which gives *k* ≈ ½(*k*_1_+ *k*_2_).

It is important to emphasize that the intent of this study was not only to estimate the time required for the complete quercetin release from nanocarriers, but also to investigate and demonstrate how the stationary and alternating field affect the rate of quercetin release. In our previous work [35], we have shown how the simultaneous application of stationary and alternating field can accelerate the release of drug from aggregates of MNPs. Being relatively unstable and dysfunctional, aggregates vigorously moved under the influence of the alternating field which resulted in drug release enhancement. MNPs that were additionally functionalized and stabilized by the PEG layer were used for this purpose in the present study. Figure 8 shows the release kinetics of quercetin at the temperature of 30 °C in the absence of the magnetic field and under an alternating field of 10 kHz, 50 kHz and 100 kHz at a constant stationary magnetic field *B* = 11 mT.

Since we used a dialysis membrane bag, the first step was to perform calibration experiments with free quercetin following the same protocol as in experiments with MNPs to get information about membrane permeation kinetics during the first several hours (*k* = 6.617 × 10^−3^ min^−1^; *k* = 9.637 × 10^−3^ min^−1^ and *k* = 14.592 × 10^−3^ min^−1^ at 25 °C, 30 °C and 37 °C, respectively). We selected the MWCO cellulose membrane (8 kD) membrane based on the porosity of the dialysis membrane as well as to avoid possible adverse interactions between quercetin and the membrane materials. The cellulose membrane has recently been used in the measurement of both, drug diffusion and drug release rates from varied formulations, such as creams and hydrogels [7].

The rate constant of the membrane when there was non-saturated quercetin solution in the membrane bag indicated the barrier effects of dialysis membrane and showed faster membrane permeation kinetics at higher temperatures (*k* = 0.0066 min^−1^, 0.0096 min^−1^ and 0.0146 min^−1^ at 25 °C, 30 °C and 37 °C, respectively), as expected. The rate constants obtained in our experiments are larger than those obtained in release kinetics of doxorubicin by Yu et al., where *k* = 0.019 ± 0.003 *h*^−1^ = 0.0003 min^−1^ [60].

With no magnetic field and at 30 °C, the rate constant is *k* = 0.0019 ± 0.0001 min^−1^. At 10 kHz, 50 kHz and 100 kHz and under *B* =11.0 mT the rate constant values were *k* = 0.0032 ± 0.0009 min^−1^, *k* = 0.0019 ± 0.0001 min^−1^ and *k* = 0.0034 ± 0.0013 min^−1^, respectively. Thus, the release of the quercetin is the faster at the highest field frequency. It is evident that the alternating magnetic field can accelerate the quercetin release at a given stationary magnetic field.

Our next task was to see the effect a stationary magnetic field on release kinetics. Figure 9 shows the dependence of quercetin-release kinetics at 25 °C under the alternating field frequency of 10 kHz and stationary magnetic field of *B* = 7.9 mT and 11 mT (Figure 9C). Under the stationary magnetic field of 7.9 mT, the membrane bag has released quercetin with a rate constant *k* = 0.0043 ± 0.0003 min^−1^. When a stronger stationary field of 11 mT is applied, the release kinetics becomes slower, *k* = 0.0024 ± 0.0012 min^−1^. The opposite effect on the release kinetics was observed at the highest 100 kHz frequency (Figure 9A), where an increase in the rate constant with increasing stationary field by almost 95% was observed (at *B* = 7.9 mT and *B* = 11 mT, *k* = 0.0015 ± 0.0002 min^−1^; *k* = 0.0029 ± 0.0004 min^−1^, respectively).

Since the opposite effects of the influence of a stationary magnetic field on the rate constant are obtained at different frequencies of the alternating field, our next analysis focuses on the measurements of the rate constants at the same stationary field and frequency, but at different temperatures. Comparing the constants of the apparent release rate of quercetin at a stationary field of 7.9 mT with an increase in temperature from 25 to 37 °C (*k* = 0.0022 ± 0.0004 min^−1^, *k* = 0.0022 ± 0.0008 min^−1^ and *k* = 0.0025 ± 0.0002 min^−1^ for 25 °C, 30 °C and 37 °C, respectively), it can be seen that the rate constant slightly increases with increasing temperature only above 30 °C. The same effect was observed under the stronger stationary field (*B* = 11 mT, see Table 2). At higher temperatures kinetic energy of quercetin molecules is larger or, putting differently, their diffusivity is larger and more quercetine is released and detected within the same time interval. If we compare the rate constants at the same temperature (e.g., 30 °C), the rate constant obtained at a stronger stationary field (*B* = 11 mT) and 50 KHz, is smaller (*k* = 0.0019 ± 0.0001 min^−1^) than at a weaker field of 7.9 mT (*k* = 0.0022 ± 0.0008 min^−1^). It is obvious that when overcoming stationary field, the thermal energy of the MNPs is large enough to increase the movement of the MNPs and increase quercetin release. The influence of the temperature under the constant stationary field and the frequency of the alternating field clearly shows that the amount of quercetin released increases with increasing temperature at almost all applied frequencies of the alternating fields except at *B* = 11 mT and frequencies *f* = 100 and 50 kHz and at *B* = 7.9 mT and *f* = 50 kHz, which is also reflected in the magnitudes of the quercetin release rate constants. For example, at *B* = 11 mT and *f* = 100 kHz, *k* = 0.0029 ± 0.0004 min^−1^; *k* = 0.0034 ± 0.0013 min^−1^ and *k* = 0.0038 ± 0.0013 min^−1^ for 25 °C, 30 °C and 37 °C, respectively.

Thus, it was shown here that by choosing the temperature, the quercetin release rates can cover a wide range of values. We have shown that the synthesized MNPs are suitable nanocarriers for quercetin, especially because the required drug dose can be delivered in a prolonged time. Since the average half-life of quercetin absorbed in the human body is 3.5 h [61], this study represents a significant improvement for flavonoid delivery, which, when loaded into MNPs, remains stable in a prolonged period of time.

## 4. Conclusions

In summary, superparamagnetic magnetite nanoparticles (MNPs) were prepared by solvothermal method and stabilized by biocompatible poly (ethylene glycol) PEG-4000 Da. The X-ray powder diffraction patterns of the synthesized bare and PEG-coated MNPs confirmed the cubic inverse spinel structure of MNPs. The size and morphology of bare and PEG-coated MNPs have been obtained by SEM, TEM and AFM analysis. By AFM is showed that bare MNPs have cluster structure with a very rough surface and when bare MNPs is coated with PEG, the roughness of the MNPs surface has been increased by PEG coating, for almost 100%, from 5.58 ± 1.06 nm to 10.9 ± 2.1 nm, confirming the effective coverage of the bare MNPs by PEG. A size histogram of bare mesoporous MNPs obtained using SEM micrographs shows a broader size distribution than PEG-coated MNPs (*d*_ave_ = 103.4 ± 0.7 nm and 101.0 ± 0.9 nm for bare and PEG-coated MNPs, respectively), indicating that PEG decreased the magnetic interaction among the particles and prevent their agglomeration. Nitrogen adsorption–desorption of bare MNPs and PEG-coated MNPs confirmed their mesoporosity. The PEG molecules were successfully coated on the surface of MNPs, as revealed by FTIR spectroscopy. The PEG-coated MNPs spectrum showed a strong C-O-C ether stretch at 1110 and 1381 cm^−1^. The results of the saturation magnetization confirmed the superparamagnetic properties of synthesized bare and PEG-coated MNPs. The stability of MNPs improved after PEG modification, indicated by the increase in zeta potential from (−30.6 ±0.7) mV to (−35.1 ± 1.5) mV.The loading of quercetin into MNPs was confirmed by FTIR spectroscopy and thermogravimetric analysis. The UV/VIS spectra of the supernatant revealed a loading efficiency of (20.2 ± 1.3%). The quercetin release studies in vitro followed by UV/VIS spectroscopy have shown the prolonged quercetin-release kinetics from MNPs that could be controlled by using combined stationary and alternating magnetic fields. The prolonged quercetin release, as an important characteristic for targeted drug delivery, the study of the kinetic parameters of the quercetin release process and the increased response of quercetin release under both the lower stationary magnetic field (7.9 mT) and the higher frequency of alternating magnetic field (100 kHz) suggest that the fine tuning of the release as desired along with synergism of physicochemical and superparamagnetic properties enables the great potential of MNPs as a promising targeted flavonoid delivery system.

## Figures and Tables

**Table 1:** Zeta potential and hydrodynamic diameter of bare MNPs, PEG-coated MNPs and quercetin-loaded MNPs.

	Bare MNPs	PEG Coated MNPs	Quercetin Loaded PEG_MNPs
*ζ*/mV	−30.6 ± 0.7	−35.1 ± 1.5	−31.3 ± 0.8
*d_h_*/nm	877 ± 105	762 ± 92	681 ± 73
PDI	0.54 ± 0.10	0.47 ± 0.10	0.52 ± 0.07

**Table 2:** The experimental release kinetics under the permanent magnetic fields of 7.9 mT and 11.0 mT and three frequencies (10 kHz, 50 kHz and 100 kHz) at temperatures 25 °C, 30 °C and 37 °C.

*B*/mT	*f*_alt.mag.f._/kHz	*θ/*°C	*k*/min^−1^	*m*_0_/mg
11.0	100	25	0.0029 ± 0.0004	0.82 ± 0.01
		30	0.0034 ± 0.0013	1.38 ± 0.30
		37	0.0038 ± 0.0013	1.88 ± 0.25
	50	25	0.0022 ± 0.0004	1.03 ± 0.30
		30	0.0019 ± 0.0001	1.41 ± 0.01
		37	0.0025 ± 0.0015	3.3 ± 1.4
	10	25	0.0024 ± 0.0012	1.07 ± 0.34
		30	0.0032 ± 0.0009	0.97 ± 0.20
		37	0.0033 ± 0.0003	1.73 ± 0.22
7.9	100	25	0.0015 ± 0.0002	1.79 ± 0.14
		30	0.0032 ± 0.0003	1.15 ± 0.44
		37	0.0052 ± 0.0011	1.50 ± 0.39
	50	25	0.0022 ± 0.0007	1.20 ± 0.26
		30	0.0022 ± 0.0008	1.86 ± 0.35
		37	0.0025 ± 0.0002	1.21 ± 0.01
	10	25	0.0043 ± 0.0003	1.13 ± 0.08
		30	0.0021 ± 0.0006	1.30 ± 0.24
		37	0.0016 ± 0.0005	1.64 ± 0.52
0	0	25	0.0022 ± 0.0004	1.03 ± 0.30
		30	0.0019 ± 0.0001	1.41 ± 0.01
		37	0.0025 ± 0.0015	2.25 ± 1.44

## Data Availability

Data available on request due to restrictions e.g., privacy or ethical. The data presented in this study are available on request from the corresponding author. The data are not publicly available due to extreme quality of data.
